# Analysis of the discrepancies identified during medication reconciliation on patient admission in cardiology units: a descriptive study

**DOI:** 10.1590/1518-8345.0820.2760

**Published:** 2016-08-15

**Authors:** Natália Fracaro Lombardi, Antonio Eduardo Matoso Mendes, Rosa Camila Lucchetta, Wálleri Christini Torelli Reis, Maria Luiza Drechsel Fávero, Cassyano Januário Correr

**Affiliations:** 1 Master's Student, Departamento de Farmácia, Universidade Federal do Paraná, Curitiba, PR, Brazil.; 2 Master's Student, Departamento de Clínica Médica, Universidade Federal do Paraná, Curitiba, PR, Brazil.; 3 Doctoral Student, Departamento de Farmácia, Universidade Federal do Paraná, Curitiba, PR, Brazil. Professor, Departamento de Farmácia, Universidade Federal do Paraná, Curitiba, PR, Brazil.; 4 PhD, Adjunct Professor, Departamento de Farmácia, Universidade Federal do Paraná, Curitiba, PR, Brazil.

**Keywords:** Medication Reconciliation, Patient Safety, Medication Errors.

## Abstract

**Objectives:**

this observational study aimed to describe the discrepancies identified during medication reconciliation on patient admission to cardiology units in a large hospital.

**Methods:**

the medication history of patients was collected within 48 hours after admission, and intentional and unintentional discrepancies were classified as omission, duplication, dose, frequency, timing, and route of drug administration.

**Results:**

most of the patients evaluated were women (58.0%) with a mean age of 59 years, and 75.5% of the patients had a Charlson comorbidity index score between 1 and 3. Of the 117 discrepancies found, 50.4% were unintentional. Of these, 61.0% involved omission, 18.6% involved dosage, 18.6% involved timing, and 1.7% involved the route of drug administration.

**Conclusion:**

this study revealed a high prevalence of discrepancies, most of which were related to omissions, and 50% were unintentional. These results reveal the number of drugs that are not reincorporated into the treatment of patients, which can have important clinical consequences.

## Introduction

According to the World Health Organization (WHO), patient safety involves reducing the risk of unnecessary harm to health to the minimum level acceptable^1^. Medication errors are considered the primary reason for harm to the health of hospitalized patients and can occur in any drug therapy stage, from prescription to administration^2^ ^-^ ^5^. More than 50% of medication errors occur when patients are discharged or transferred between units, indicating that the transition stages are prone to the occurrence of errors^6^. 

A thorough and accurate medication history should be obtained at the time of drug prescription to increase drug safety^7^ ^-^ ^8^. Up to 27% of all prescription errors are related to incomplete medication histories at the time of admission, leading to discrepancies between the drugs used before admission and those used during hospitalization. Previous studies have indicated that 60 to 70% of medication histories contain at least one error, and 59% of all errors have a major clinical impact^8^ ^-^ ^11^. The collection of an accurate medication history at the time of patient admission is essential to guarantee patient safety. The incorrect collection of medication history is responsible for most of the adverse drug reactions experienced after hospital discharge and can compromise the continuity of treatment^11^ ^-^ ^12^. 

Previous studies have shown that medication reconciliation at the time of patient admission decreases the number of discrepancies between the drugs used before admission and those prescribed during hospitalization^8^ ^,^ ^10^ ^,^ ^13^ ^-^ ^14^.

The objective of this study was to describe the discrepancies found in medication reconciliation on patient admission to a clinical cardiology unit, a chest pain unit, and a coronary care unit of a large hospital. 

## Methods

This cross-sectional, descriptive study was conducted in a large university hospital. The data presented in this study are part of a randomized clinical trial that was conducted between May 2013 and January 2014 in five clinical units of the hospital. In the randomized clinical trial, the calculated sample size was 65 patients per group to achieve a detection power of 80% for two predetermined outcomes: length of stay and mortality. 

All of the patients admitted to the clinical cardiology unit, chest pain unit, and coronary care unit were identified prospectively by a clinical pharmacist between May 2013 and January 2014. The patients admitted on weekends were identified on the first working day after admission. 

The study included patients aged ≥18 years who were admitted to one of the selected hospital units and who agreed with the criteria outlined in the free and informed consent form. Patients were excluded for the following reasons: their medication histories were not collected in the first 48 hours after admission, they were discharged before collection of the medication history, they had already been included in a previous study, they were admitted before the study period, and they could not provide the information necessary for the study because of impaired cognition, being under mechanical ventilation, or lacking a caregiver who could help in data collection.

This study was approved by the Ethics Committee of the Clinical Hospital of the Federal University of Paraná under Protocol no. 14179613.7.0000.0096.

## Drug reconciliation

Medication history was collected via interviews with the patient or caregiver, considering the best possible history developed according to previous recommendations^15^ ^-^ ^16^based on combining information from the community pharmacy record, the information provided by a structured interview with participants about their medication use, and medication containers. In nine hospitals, pharmacy technicians obtained the BPMH, and in three hospitals, a mixed model was used (physicians or pharmacy technicians obtained the BPMH, and via assessment of the patient records to complement the medication history data. The data were collected using the following potential sources of information: patient, drug prescriptions, drugs brought from home, bedside charts, family or caregiver, and information provided by municipal health units and health care institutions or living facilities. 

After data acquisition, a list of pre-admission medications was developed and then compared with the medications prescribed on patient admission. This comparison allowed the identification of discrepancies between the two lists, defined as any differences between the medication history collected and the medications prescribed to the patient on admission^10^.

**Figure 1:**
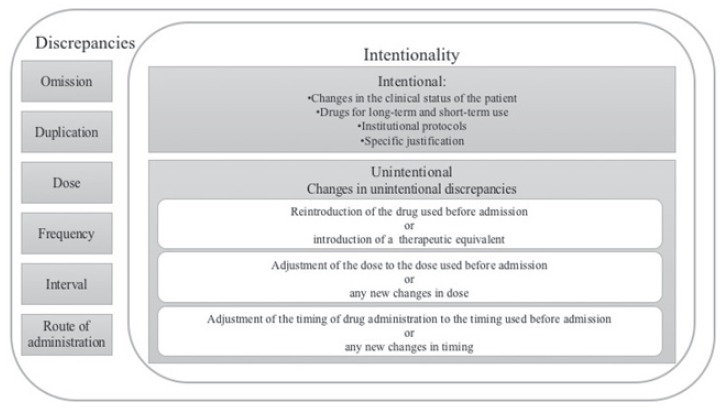
Classification of discrepancies according to type, intentionality, and changes made to correct the discrepancies.

The discrepancies were classified according to type, intentionality (intentional or unintentional), and changes made by the physician during hospitalization (Figure 1)^8^ ^,^ ^13^.

During the hospitalization period, the unintentional discrepancies corrected by the physician were classified according to the following factors: I) reintroduction of medications used before admission or introduction of a therapeutic equivalent; II) adjustment of the dose to the dose used before admission or any new changes in dose; or III) adjustment of the timing to the timing used before admission or any new changes in timing.

## Results

During the eight-month study period, 229 patients were admitted to the selected units. Of these, 24 patients were included in the conciliation service, and 202 patients were excluded for the following reasons: discharge or transfer to another unit before the medication history was collected (n = 9), hospitalization for more than 48 hours without collection of medication history (n = 162), death before conciliation (n = 3), communication impairment and lack of a caregiver to help in data collection (n = 7), admission for elective surgery (n = 7), and refusal to participate in the study (n = 14). 

The study group was primarily composed of women (58.0%) with a mean age of 59 ± 6.0 years; the participants were admitted for various clinical conditions. The most frequent comorbidities were associated with the cardiovascular and endocrine systems, including hypertension (79.0%), coronary artery disease (54.0%), dyslipidemia (50.0%), and diabetes mellitus (33.0%). The Charlson comorbidity index (CCI) was used to assess the risk of death of the patients for the following ten years (Table 1).

**Table 1:** Characteristics of the study population. Curitiba, state of Paraná, Brazil, 2014

Patient characteristics		Study population	
		n = 24	%
Age in years, mean ± SD*		59 ± 6	
Gender			
	Women	14	58.3
Independence in the management of pharmacotherapy			
	Patient	23	95.8
	Caregiver	1	4.2
CCI^†^			
	0	3	12.5
	1	9	37.5
	2	4	17.0
	3	5	21.0
	4	2	8.0
	5	0	0
	6	1	4.0
Days of hospitalization, median (IQR^‡)^		15 (8-19)	

*SD: standard deviation; †CCI: Charlson comorbidity Index; ‡IQR: interquartile range

In most cases (42%), the patients were the only source of information. For 37% of the patients, two sources were consulted; for 17% of the patients, three sources were consulted; and for 4% of patients, four sources were consulted. Half of the patients (50%) brought their medications to the hospital on admission (or the caregiver brought them later), 29.0% brought a list of drugs, and in 4.0% of cases, the caregiver or family member helped collect and disclose history data. 

The drugs involved in intentional and unintentional discrepancies were classified according to the Anatomical Therapeutic Chemical (ATC) classification system, and the drug groups with the highest prevalence were those used to treat complications in the cardiovascular system (n = 43), nervous system (n = 13), gastrointestinal tract and metabolism (n = 11), and blood and blood-forming organs (n ​​= 10).

In addition, 217 prescription drugs were identified on admission, and of these, 53.9% (n = 117) were involved in the discrepancies. In total, 58 (49.6%) discrepancies were intentional, and 59 (50.4%) were unintentional (Table 2). 

**Table 2:** Types of intentional and unintentional discrepancies identified. Curitiba, state of Paraná, Brazil, 2014

Type of discrepancy	Intentional n (%)	Unintentional n (%)	Total n (%)
Omission	48 (82.8)	36 (61.0)	84 (71.8)
Dose	10 (17.2)	11 (18.6)	21 (18.0)
Timing of administration	0	11 (18.6)	11 (9.4)
Route of administration	0	1 (1.7)	1 (0.8)
Total	58 (100.0)	59 (100.0)	117 (100.0)

Among the unintended discrepancies, in 20.3% (n = 12) of cases, the omitted drug was reintroduced during hospitalization, or the medication was prescribed again with changes in the dose, route, or timing of administration in relation to pre-admission. In addition, in 37.3% (n = 22) of these discrepancies, a therapeutic equivalent was included in the drug prescription to replace the drug involved in the discrepancy, or changes were made to the dose, route, or timing of administration (Table 3). 

**Table 3:** Types of unintentional discrepancies and whether drug therapy was reintroduced with or without changes. Curitiba, state of Paraná, Brazil, 2014

Type of discrepancy		Frequency of each discrepancy (%)	Total number of discrepancies in each reintroduction category
Reintroduction of the medication without changes			12
	Omission	75.1	
	Dose	8.3	
	Timing	8.3	
	Route	8.3	
Reintroduction of the medication with changes			22
	Omission	9.0	
	Dose	45.5	
	Timing	45.5	
	Route	0	

## Discussion 

Discrepancies in medication history may impair the effectiveness and safety of drug therapy. In this study, 54.0% of the medication histories presented some type of discrepancy. The most common discrepancies were omission (medications used before admission but not prescribed during hospitalization) and dose differences between pre-admission and hospitalization. Similar studies corroborate this result, particularly with respect to the higher incidence of omissions^8^ ^,^ ^10^ ^-^ ^11^ ^,^ ^13^ ^,^ ^17^ ^-^ ^18^.

The number of intentional and unintentional discrepancies differed between studies. A previous study found 866 discrepancies on admission, 93% of which were unintentional, whereas in another study, 94% of unintentional discrepancies were seen, and after interventions performed by pharmacists, 97% of the discrepancies became intentional. By contrast, a similar percentage of intentional and unintentional discrepancies was found in this study. This variation in the results can be explained by the different criteria chosen in each study to classify intentional and unintentional discrepancies, which makes the study models heterogeneous and limits data comparison. In addition, these studies elected complementary parameters, such that the first study considered intentional discrepancies to be the changes made based on the new clinical status of the patient, and the second study added two other criteria: drug replacement based on guidelines and any changes made in the route, timing, or dose^13^ ^-^ ^14^. In our study, the changes made based on the new clinical status of the patient and drug replacement based on guidelines were considered intentional discrepancies, whereas changes in route, timing, and dose of administration were found to be unintentional discrepancies; these distinct classifications may explain the differences in the results.

In our study, among the unintentional discrepancies, omission was the most prevalent. The omission of drugs upon admission may cause discontinuation of drug therapy and impair the health of the patient(19). The predominance of omissions may be related to the collection of incomplete and inaccurate medication histories. 

Among the unintentional discrepancies, in 20.3% of cases when the reintroduction of the drug therapy used before admission was necessary, the drug involved in the discrepancy was prescribed again using the same conditions used before admission, and most (75.1%) of the discrepancies identified were omissions. In 37.3% of cases, a therapeutic alternative to the drug involved in the discrepancy was included in the prescription; alternatively, a dose, route, or timing of administration different from that used before admission was used during hospitalization. In these cases, the most frequent discrepancies were dose and timing (45.5%). 

These results indicate that the drugs involved in the unintentional discrepancies were essential to the patient during hospitalization. These drugs were prescribed in the exact form in which they were used before admission, or as therapeutic equivalents, or with a dose different from that used before admission. Previous studies have found that patients with discrepancies on admission are subjected to more medication errors and medication errors upon hospital discharge, and these errors on discharge arise from discrepancies related to incomplete medication history^17^ ^,^ ^20^ ^-^ ^21^.

In 42% (n = 25) of cases of unintentional discrepancies, the omitted drugs were not reintroduced or were introduced with a dosage different from that used before admission, and this affected the ongoing treatment or even the treatment after discharge for chronic conditions. This strategy burdens the healthcare system because of the need to return to health care facilities for the treatment of complications caused by these discrepancies.

The first level of classification established by the ATC indicated that the drug groups with the highest prevalence were those used for the treatment of complications in the cardiovascular system, nervous system, gastrointestinal tract and metabolism, and blood and blood-forming organs. These results are similar to those of other studies, considering the predominance of older individuals in these studies, including ours^10^ ^,^ ^14^ ^-^ ^15^ ^,^ ^19^ ^,^ ^22^.

Some limitations should be considered in our study, including patient evaluation in only three cardiology units-clinical cardiology, chest pain, and coronary care- resulting in a small sample size. For this reason, this study is not representative of the entire healthcare system, and our results should be interpreted with caution. Nevertheless, it is believed that our study is relevant because it provides a local epidemiological profile and allows robust evaluations in the future. The structure of the healthcare services was also a limitation because only three professionals performed the medication reconciliations, and these professionals were not exclusively dedicated to conducting this activity, limiting the number of patients enrolled. In this respect, six new professionals trained to perform medical reconciliation would be required to include all of the patients admitted to the hospital in the study. Another limitation was that missing data in the medical records limited data collection^23^ ^-^ ^24^.

## Conclusion

The present study revealed a high prevalence of discrepancies, most of which were related to drug omissions. In addition, approximately 50% of the discrepancies were classified as unintentional, and most of the discrepancies were related to medications required by patients and/or drugs not reintroduced during admission. These discrepancies may cause impairments to the effectiveness and safety of patient treatment, including interruptions in the treatment of chronic conditions and a higher probability of aggravation of untreated comorbidities. 
